# Understanding Contextual Spillover: Using Identity Process Theory as a Lens for Analyzing Behavioral Responses to a Workplace Dietary Choice Intervention

**DOI:** 10.3389/fpsyg.2019.00345

**Published:** 2019-03-01

**Authors:** Caroline Verfuerth, Christopher R. Jones, Diana Gregory-Smith, Caroline Oates

**Affiliations:** ^1^Management School, University of Sheffield, Sheffield, United Kingdom; ^2^Environmental Psychology Research Group, School of Psychology, University of Surrey, Guildford, United Kingdom; ^3^Newcastle University Business School, Newcastle University, Newcastle upon Tyne, United Kingdom

**Keywords:** contextual spillover, identity process theory, behavior change, workplace, identity

## Abstract

Spillover occurs when one environmentally sustainable behavior leads to another, often initiated by a behavior change intervention. A number of studies have investigated positive and negative spillover effects, but empirical evidence is mixed, showing evidence for both positive and negative spillover effects, and lack of spillover altogether. Environmental identity has been identified as an influential factor for spillover effects. Building on identity process theory the current framework proposes that positive, negative, and a lack of spillover are determined by perceived threat of initial behavior and identity process mechanisms evaluating the behavior. It is proposed, that an environmental behavior change intervention may threaten one's existing identities, leading to either (a) integration, (b) compartmentalization, or (c) conflict between one's environmental identity and non-environmental identities. Initial evidence for the proposed framework is based on a field intervention which included a meat reduction programme in a canteen of a medium size private sector company. Semi-structured interviews and an explorative visualization method that aimed at assessing identity change were implemented with thirteen employees (i.e., intervention participants) before and after the intervention. The qualitative data was analyzed by using thematic analysis via NVivo12. Results of the visualization task and interview method provided initial evidence of direct and indirect positive contextual spillover effects, with comparatively less evidence a lack of spillover and a relative absence of reported negative spillover. This paper provides a novel theoretical approach, centered on identity process theory to enhance understanding of positive spillover, negative spillover, and the lack of spillover.

## Introduction

Environmental spillover effects occur when the performance of one environmentally sustainable behavior (ESB) leads to a secondary behavior being performed (Nash et al., 2017). The secondary behavior can be in the same direction as the initial behavior (i.e., *positive* spillover) or in the opposite direction (i.e., *negative* spillover) (Thøgersen and Ölander, 2003). Equally, a lack of spillover can occur where there is an absence of either positive or negative spillover effects. While there are many different definitions of spillover, we focus on a popular definition that looks at behavior change in responses to an intervention, in which spillover is defined as “the effects of an intervention on subsequent behaviors not directly targeted by it” (Truelove et al., 2014, p. 127).

Whereas the presence and encouragement of positive spillover is clearly desirable for those wishing to promote greater consistency in people's ESBs; the absence of positive spillover or, more worryingly, the presence of negative spillover is clearly less desirable (Carrico et al., 2015). The perceived importance of promoting positive spillover and restricting negative spillover within the context of ESBs has led to growing interest in the study of spillover effects (for an overview see e.g., Nash et al., 2017). Interestingly, the findings in the extant literature present a mixed picture about the phenomenon, with evidence of both positive and negative spillover (and a lack of spillover) under different conditions.

For example, in relation to positive spillover, Van der Werff et al. (2014) found that people's past ESBs were positively related to other, different ESBs at a later time. Similarly, Steinhorst et al. (2015) found empirical evidence for positive spillover between electricity saving behaviors and other climate-friendly behavioral *intentions*. Midden et al. (2007) and Klöckner et al. (2013) claim to have identified evidence of negative *behavioral* and *motivational* spillover, respectively. By comparison, Midden et al. (2007) found that people believed that the negative environmental effects of driving to work, could be compensated for by not owning a tumble dryer (pro-environmental behavior), while Klöckner et al. (2013) found that buyers of electric cars had significantly lower motivations to engage in other pro-environmetnal behaviors than buyers of conventional combustion engine cars. Yet other research has reported upon the simultaneous co-occurrence of positive and negative spillover effects (Lacasse, 2016) or a lack of spillover altogether (Poortinga et al., 2013). For example, Poortinga et al. (2013) found that the introduction of a carrier bag charge in Wales, while strengthening people's environmental identity and prompting a reduction in single-use carrier bags, did not prompt change in other waste-related behaviors.

Research into spillover is still in its relative infancy and a number of knowledge gaps still exist. For example, while there have been attempts to explain spillover effects through a theoretical lens (e.g., Truelove et al., 2014; Dolan and Galizzi, 2015), there still exists a lack of conceptual clarity over the phenomenon. Even within the studies outlined above, spillover has been conceptualized as changes in non-target (a) behaviors, (b) intentions, and (c) motivations, respectively. Moreover, much of the extant evidence of spillover has beeni based upon the findings of correlational studies, where attribution of *causality* is limited, and laboratory experiments, where real-world implications are limited. Hence, academics increasingly point to the importance of “real-world” settings when examining spillover effects and to examine the *causal* processes underpinning spillover (Sintov et al., 2017; Verfuerth and Gregory-Smith, 2018).

The majority of research conducted to date has focussed on understanding the roots of positive spillover (as opposed to negative or a lack of spillover) within one behavioral context (e.g., at home). This means that there is currently a relative lack of research investigating cross-contextual effects. This is despite contextual spillover, in addition to cross-behavioral and temporal spillover, being a recognized phenomenon warranting investigation (Nilsson et al., 2017). Of particular interest to the current article is the study of contextual spillover and, more specifically, the presence (or absence) of spillover between the workplace and home. People spend a large amount of their day-to-day lives at work and at home making the behavior within and between both contexts crucial to living sustainably (Cox et al., 2012). Despite this, however, spillover between these two settings has to date received little attention (e.g., Littleford et al., 2014).

Previous research demonstrates that identity is one of the driving factors underlying spillover effects (e.g., Whitmarsh and O'Neill, 2010); however, the consideration of how identity processes might map to all spillover variations (i.e., positive, negative and a lack of spillover) is under investigated has yet to be made. We feel that this necessitates further research into the psychological underpinnings of spillover (or the absence thereof) and thus, within this paper, outline an integrated framework of spillover based upon Identity Process Theory (Breakwell, 1986). This framework seeks to shed light on the underlying identity processes that may lead to presence or absence of spillover effects. We then present empirical findings of exploratory work to provide an “initial” test of the assumptions of our theoretical framework. The framework presented in this paper makes a novel contribution to the extant literature by proposing a route via which the presence or absence of changes of one's pro-environmental identity may (or may not) lead to spillover effects. The remainder of the introduction outlines what is currently known about the relationships between identity and spillover before introducing the conceptual model that is central to our research.

### Identity and Spillover

The way in which we see ourselves—our identity—helps us to be consistent in our behaviors across time and contexts (Whitmarsh and O'Neill, 2010). Accordingly, environmental identity (i.e., how we see ourselves in relation to the natural world) has been found to be an influencing factor for environmental actions (Clayton and Opotow, 2003) and spillover effects. For example, Lacasse (2016) found that reminding people of past environmentally sustainable behaviors and labeling them as “environmentalists” led to stronger environmental self-identity, which increased positive spillover effects. Similarly, Van der Werff et al. (2014) found that reminding people of past environmentally sustainable behaviors strengthened their environmental self-identity, which in turn led to positive spillover effects.

There is much less evidence of links between identity and negative spillover effects. One experimental study that has investigated the relationships, though, found that environmental identity mediated spillover between recycling behavior and support for a green fund among a sample of U.S. students, however, engaging in recycling behavior had a negative impact on their green identity, which in turn lowered the support for a green fund (Truelove et al., 2016). In essence, Truelove et al. (2016) suggested that students with stronger green identities (i.e., the Democratic Party supporters) were likely to view recycling behavior as an easy or mundane pro-environmental act. As such, this intervention failed to enhance the green identities of this group and thus failed to increase their support for the “green fund.”

In sum, evidence points to identity (and in particular environmental identity) as being potentially important underlying factor of environmental spillover effects. To date, though, a model of the identity-related processes that may lead to the emergence of positive *and* negative spillover effects (or a lack thereof) is noticeably lacking. We argue that Identity Process Theory (IPT, Breakwell, 1986; Jaspal and Breakwell, 2014) offers a suitable lens through which to analyse the identity-related mechanisms that might mediate the relationships between the performance of an initial environmentally sustainable behavior (e.g., following a persuasive appeal) and the emergence (or absence) of subsequent congruent or incongruent behaviors (i.e., spillover effects).

#### Identity Process Theory (IPT)

We are constantly exposed to life transitions and changes in our physical and social environment. IPT seeks to explain how these changes affect the way we think about ourselves and how individuals, in times of change, may integrate changes into their identity or, when changes are experienced as a threatening, cope with such changes (Amiot and Jaspal, 2014). IPT seeks to explain the changes that occur to one's identity in response to “threat” by examining the dynamics of social structure (e.g., society and expectations), social relationships (e.g., family) and the self-concept (i.e., ideas about the self; Breakwell, 1986; Baumeister, 1999; Amiot and Jaspal, 2014).

Two processes are thought to regulate one's identity: the process of assimilation-accommodation and the process of evaluation (Breakwell, 1986). The process of assimilation-accommodation refers to how new information is absorbed into one's self-concept and the adjustment that occurs in one's self-concept as this happens. During the assimilation-accommodation process, the goal is to maintain or modify the existing self-identity by integrating new information (e.g., new knowledge, attitudes, beliefs, or behavior) into the existing self-concept either by integrating information into existing identity structure (i.e., assimilation) or by making changes to the identity structure (i.e., adaptation). During the evaluation process, the individual attains meaning and value to the contents of one's self-identity and aims to achieve a balance in one's sense of self (Jaspal and Breakwell, 2014).

Four key principles guide these two processes: (1) continuity; (2) distinctiveness; (3) self-efficacy; and (4) self-esteem. Similar to the tenets of cognitive dissonance theory (Festinger, 1957), the principle of continuity suggests that people have a desire to maintain consistency. This drives them to maintain a consistency in their identity across contexts and time (Jaspal and Breakwell, 2014). The distinctiveness principle drives people to maintain a uniqueness or distinctiveness of character from others. While the principles of self-efficacy and self-esteem drive people to maintain a sense of perceived control over their lives and a feeling of self-worth, respectively. It is the interplay between the processes of assimilation-accommodation and evaluation, and these four guiding principles which, according to IPT, can lead to the presence or absence of a change in identity over time (Jaspal and Breakwell, 2014).

IPT asserts that where conflict arises between the universal processes and the guiding principles, for whatever reason, a person's identity is threatened, and this will activate intrapersonal (e.g., denial), interpersonal (e.g., isolation from others), and/or intergroup (e.g., social mobilization) coping strategies designed to resolve the threat. For example, someone who derives their sense of distinctiveness and self-worth from driving an attractive but fuel-inefficient car, could perceive persuasive attempts to reduce car use on environmental grounds to be threatening to their sense of self (Murtagh et al., 2012). This threat could be resolved in a number of ways. For example, one could seek to deny that there is an environmental issue (or their responsibility for causing the issue) and perhaps mobilize behind others who share this perception; or they might evolve their self-perception in response to the threat and alter their behavior accordingly (e.g., reduce their car use and/or purchase an attractive, fuel-efficient car to drive). According to Jaspal and Breakwell (2014) it is by examining how people respond to identity threat that one gets a sense of the processes that underpin identity construction.

### Conceptual Framework

**Figure 1:**
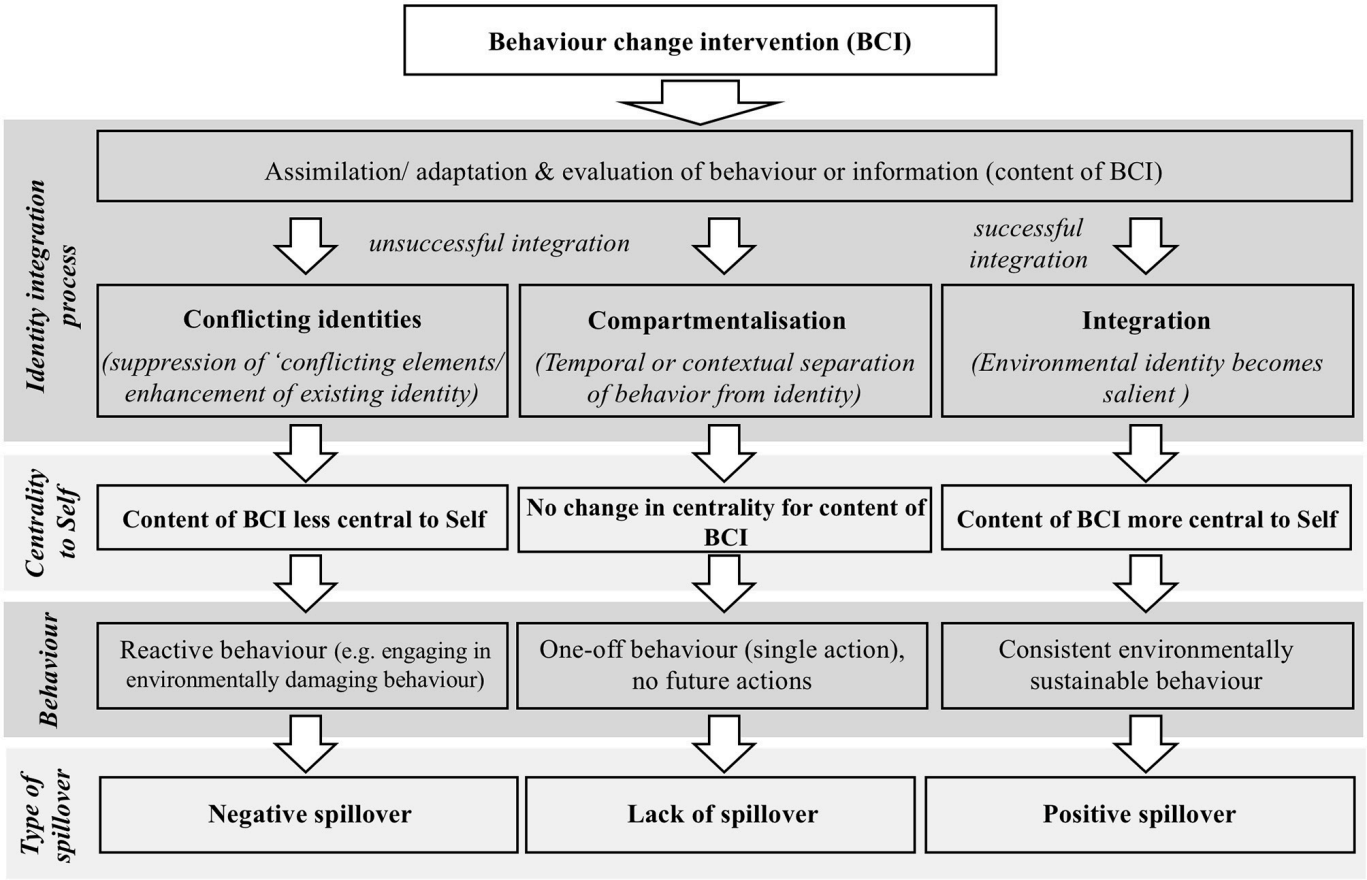
Conceptual framework for understanding spillover.

Our conceptual framework for understanding spillover (see Figure 1) operates on similar principles to IPT. In this context, we define spillover as being an observable change in an ESB caused by a change in an antecedent ESB. We argue that engaging in an ESB (e.g., triggered by environmental behavior change intervention) sets in motion a process of integration of the information into one's identity. If successful, such integration can result in positive spillover occurring but, if unsuccessful, the lack of appropriate identity integration may result in negative spillover effects (or a lack of spillover).

Using a workplace example, imagine a scenario in which an employee is exposed to an energy-saving intervention in the workplace. The person receives new information about the negative impacts that wasting energy at work can have on the environment and their options for reducing this impact. In processing this information, the person begins the process of integrating (i.e., assimilating or accommodating) the information into their existing identity structures and assessing (i.e., evaluating) the meaning this information holds for their sense of self. Where the information is deemed to fit with the four core guiding principles (e.g., the suggestions are perceptively achievable and facilitate their pro-environmental sense of self in the workplace context), the suggestions are likely to be absorbed (i.e., assimilated) and will strengthen the importance of his or her green identity—i.e., we see full *identity integration*. This hypothesis is consistent with prior research that shows how engaging in ESBs can strengthen one's environmental self-identity (e.g., Van der Werff et al., 2014) or serve to make one's pro-environmental self-identity more salient (Lacasse, 2016).

If assimilation of the information is not feasible or desirable to process the information received, for example, in a persuasive appeal, accommodation can occur. This is where one's identity structures is modified in some way in order to fit with the incoming information. For example, a person might watch a documentary about environmental and ethical issues of animal farming and decides to adopt a vegan diet. While the assimilation process strengthens one's existing identity, the adaptation process leads to qualitative changes in the identity structure.

According to our model, however, *identity integration* is not guaranteed. For instance, where the tenets of a persuasive appeal are viewed as inconsistent with one's guiding principles, we propose that one of two things will happen. Drawing on a stage model that explains the integration of multiple social identities into the self (Amiot et al., 2015), we suggest that an unsuccessful integration may lead to *compartmentalization* of identities or the emergence of *conflicting identities*.

In the case of *compartmentalization*, the individual maintains their existing self-identity by confining their response to a persuasive appeal to a particular time or context. Compartmentalization is a strategy taken to avoid the emergence of (undesirable) identity conflict (Hirsh and Kang, 2016). In terms of *temporal compartmentalization*, people will confine their response to a persuasive appeal to a particular point in time. By isolating their response to the appeal in this way, the person is likely to respond appropriately to the appeal at the time it is experienced but without any long-term changes to their identity. Thus, once the appeal is removed, the person's behavior is likely to return to how it was before the appeal. This form of compartmentalization is certainly consistent with phenomena such as the single action bias (Weber, 2006) or the tokenistic ESBs evoked by environmental behavior change interventions or mental accounting (Schütte and Gregory-Smith, 2015).

In the case of *contextual compartmentalization*, the individual compartmentalizes their identity into parts that may be context dependent. For instance, in our workplace example, our employee might separate their “workplace” identity from other aspects of their character (e.g., their identity in the home or in leisure contexts) and respond to the tenets of the appeal solely within the “workplace” context. This assertion is consistent with the principles of boundary theory in which Ashforth et al. (2000) propose that people will sometimes segment their life-roles and associated identities (e.g., separating their home and work lives) to create boundaries to help simplify and order their social world. Where people successfully isolate a context within which a persuasive appeal is received, this restricts the chances of any longer-term identity-shift or behavior change in other contexts.

Where an individual fails to successfully absorb the tenets of a persuasive appeal or where they fail to manage the threat via compartmentalization, *conflicting identities* can emerge. Within our worked example, for instance, our employee might positively respond to the tenets of the energy saving appeal (due to their sense that being pro-environmental is a good thing) but simultaneously realize that acting in accordance with the appeal might compromise their abilities to make money for the company; pride in which is central to their sense of self. In the presence of conflict identities, coping processes are activated in order to dissolve the experienced conflict (see IPT, Breakwell, 1986). For the purposes of our proposed framework, we draw specifically upon two coping mechanisms advocated by Hirsh and Kang (2016): (1) *suppression* of conflicting elements; or (2) *enhancement* of elements that are central to the individual's identity.

Where *suppression* occurs, attempts will be made to undermine or devalue one of the conflicting elements in order to resolve the dissonance. For example, the employee in our example might question the net value of the workplace energy saving campaign. In doing so, they can justify not fully engaging with the appeal, while simultaneously maintaining an economically profitable (but energy intensive) “business as usual” approach to their workplace behavior. Where *enhancement* occurs, the conflict is resolved by bolstering (rather than undermining) one of the conflicting identities. For example, our employee might seek to resolve the conflict between their pro-environmental and pro-economic identities, by inflating the perceived importance of making money for the company in spite of the recognized need to be more pro-environmental.

In sum, our conceptual model indicates that there are broadly three ways in which people might respond to an environmental persuasive appeal, which have differing implications for their identity. Where the tenets of the appeal are successfully integrated, this should strengthen one's green identity making it more central to their sense of self. Where integration is unsuccessful, however, this could lead to temporal or contextual compartmentalization or the emergence of conflicting identities. Crucially, where conflicting identities arise, this could serve to decrease the centrality of one's green identity relative to other identities.

#### Implications for Spillover

We argue that the nature of the identity *integration* that occurs in response to a persuasive appeal will have implications for spillover effects. Specifically, if integration of the tenets of the appeal is successful, we predict that this will increase the likelihood that positive spillover will occur. The strength of one's green identity is known to have implications for one's likelihood of engaging in ESBs (Whitmarsh and O'Neill, 2010). Therefore, where the centrality of one's green identity is strengthened, one should anticipate greater expressions of ESBs to follow (as people seek to act in an identity-consistent way in order to avoid dissonance) and evidence of positive spillover to occur as a result.

We hypothesize that in situations where *compartmentalization* occurs, that there will be little likelihood of spillover (i.e., a lack of spillover). This is particularly likely in the case where the compartmentalization of identity is achieved on *temporal* grounds, as people are likely to respond to the tenets of a persuasive appeal only as they are received. In the case of *contextual* compartmentalization, we anticipate that while spillover *between* contexts would be unlikely (e.g., an employee of a company trialing a workplace energy efficiency campaign would not change their household behaviors), some evidence of spillover *within* the compartmentalized context might occur (e.g., the employee might also seek to save water or reduce general consumption in the workplace).

Finally, in the case of conflicting identities, we anticipate that there are two likely outcomes for spillover depending upon the coping mechanism employed. Where suppression of one's green identity occurs, we anticipate that this will lead to a tokenistic or nil response to the persuasive appeal and an associated lack of any spillover. By contrast, where an alternative (i.e., non-green identity) is bolstered in order to resolve the conflict, we argue that this could result in a maladaptive (i.e., environmentally damaging) response to the persuasive appeal and (potentially) the emergence of negative spillover effects.

More worryingly, perhaps, there is evidence that where conflicting identities arise people can seek to engage the support of others in order to resolve the dissonance (a form of intergroup coping mechanism; Breakwell, 1986). Within the context of maladaptive responses to environmental persuasive appeals, this could mean that people will seek to mobilize others to rebel against the tenets of the appeal, further undermining its effectiveness. We argue that this is a particularly pertinent consideration within group contexts, such as the workplace.

## Study Design and Context

To test the theoretical assumptions drawn from our identity-based spillover framework, a field study was conducted in a medium size (c. 1,000 employees), private, service-sector company (i.e., internet service provider). The field study ran during the summer of 2017 and comprised a workplace behavior-change intervention (centered upon dietary choice) accompanied by a series of pre- and post-intervention qualitative interviews, observations and a survey.

The current article focuses specifically on the findings of the qualitative interviews, which were designed to probe participants' perceptions of sustainability, their identity in relation to dietary choice and to explore evidence for any contextual spillover effects from the work to the home setting resulting from the behavior change intervention. While there is still a tendency toward the use of quantitative methods within spillover research, our study joins a growing number of studies employing qualitative methods to shed light on processes driving spillover (e.g., Schütte and Gregory-Smith, 2015; Uzzell and Räthzel, 2018).

This study was carried out in accordance with the recommendations of the Research Ethics Policy of the University of Sheffield. The study protocol was approved by University of Management School ethics committee in accordance with the University of Sheffield ethics policy. All participants gave written informed consent. While the company contributed to the research project by allowing employees to take part in interviews and surveys during their working hours, no financial contribution was made.

### Materials and Methods

#### Participants and Sample Selection

Participants were recruited via a short online survey distributed via email to all employees of the partner company. This survey was distributed in advance of the planned behavior change intervention. The survey contained a number of questions designed to assess food consumption, environmental self-identity and included a “stages of change” scale (Bamberg, 2013), which was adapted to assess stages of change with respect to their transitions toward more sustainable dietary choice.

The survey also asked if participants would be willing to participate in each of two interviews, one to be held in advance of and one to be held after the intervention. Participants were informed that participation in this interview would be optional. As we were interested in understanding more about how people at different sustainable dietary stages would respond to the intervention, we screened participants' responses to this measure in order to identify a range or prospective interviewees.

The prospective interviewees (*N* = 23) were re-contacted and invited to take part the semi-structured interviews. All prospective participants were offered a £10 Amazon voucher as payment for their participation in the interviews. Of these prospective interviewees, *n* = 13 took part in both the pre- and post-intervention interviews (T1 and T2). It is these participants that constitute the sample for the following analysis.

The semi-structured interviews, each lasting between 30 and 60 min, were conducted 1 month before and after the behavior change intervention. All interviews took place in the canteen of the company. The sample comprised seven women and six men aged between 18 and 55 years (see Table 1). The interviewees' job role within the company varied but was mostly customer service or technical support related.

**Table 1:** Sample information.

**ID**	**Gender**	**Age**	**Education**	**Job role**	**Months worked at company**	**Stages of Change**
104	Female	18–25	N/A	Payment Team	8	Precontemplation
108	Female	26–35	A/AS level	Engineer	72	Contemplation
117	Male	26–35	University degree (BSc/BA)	n/a	47	Contemplation
107	Male	26–35	University degree (BSc/BA)	Operations	18	Contemplation
110	Female	36–45	University degree (BSc/BA)	Digital manager	14	Contemplation
106	Female	26–35	Master's degree	Analyst	6	Contemplation
129	Female	36–45	University degree (BSc/BA)	Customer Service	21	Contemplation
112	Female	26–35	Master's degree	Analyst	11	Preparation/ Action
105	Male	26–35	GCSE/O level	Technical support	48	Preparation/ Action
126	Female	36–45	A/AS level	Team leader	72	Maintenance
131	Male	26–35	University degree (BSc/BA)	Junior Engineer	84	Maintenance
102	Male	36–45	University degree (BSc/BA)	Software Engineer	11	Maintenance
132	Male	46–55	GCSE/O level	Sales	46	Maintenance

### Behavior Change Intervention

The behavior change intervention targeted food choice with a particular focus on reducing red meat consumption among employees. Dietary choice, especially meat consumption, is associated with considerable negative environmental impacts, with recent estimates indicating that a saving of 0.8 tons CO_2_ (equivalent) per year could be saved for every person who switches to a plant-based diet (Wynes and Nicholas, 2017). Moreover, to the extent that dietary choice is often related to identity (e.g., Bisogni et al., 2002; Fox and Ward, 2008), it became a natural target behavior for the intervention.

The company that hosted this study has a canteen in which simple meals are provided for free to the employees (e.g., sandwiches, jacket potatoes, salads provided as a buffet) and hot meals for a subsidized price. For the behavior change intervention, a new “sustainable choice” menu was developed along with the company chef. Menu options were based upon the recommendations made by the United Nations Food and Agriculture Organization (FAO) (Fischer and Garnett, 2016). Additional input into the menu design came from the results of a short employee survey and the pre-intervention interviews (for more information, see Supplementary Material A).

The sustainable choice menu reduced the quantity of available meat-based food options by 70% (relative to the normal menu and a total removal of beef or lamb) and saw an increase in the number of plant-based options, vegetables and low or non-processed foods. Each food item was assigned to an information sheet about nutrient content and ingredients (this had been provided in the canteen previously). The hot meals, which previously contained two meat options, were changed to include one vegan or vegetarian dish and one meat dish (only white meat). To increase the acceptance of the menu changes, all employees were invited to give feedback to the menu (see Data Sheet 1). The menu changes were implemented for 1 week in the summer of 2017.

**Figure 2:**
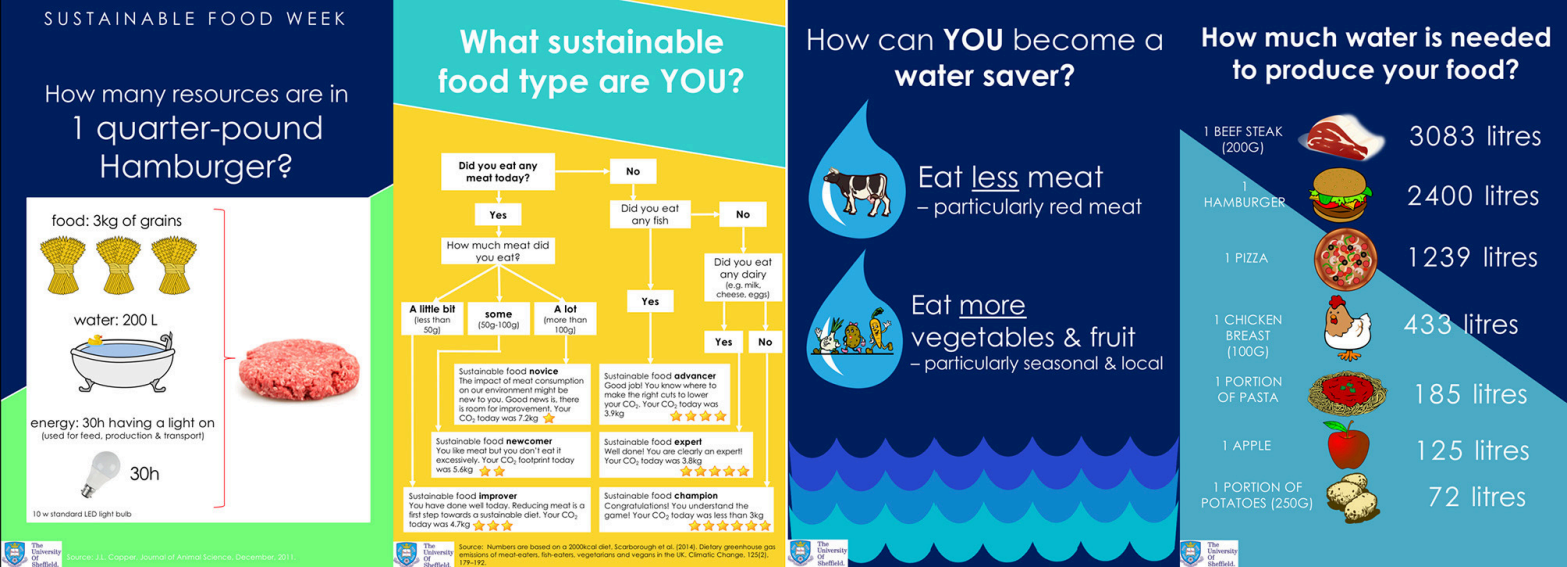
Examples of information material used in behavior change intervention.

This new menu was delivered as part of a broader information campaign, which sought to raise awareness of the impacts of food choice (in terms of CO_2_ emissions, water use and land use), as well as including normative messaging. The information was delivered in the form of posters, that were hung in obvious places within the canteen and “table talkers” placed upon each table within the canteen (see Figure 2).

### Semi-structured Interviews and Visualization

Semi-structured interviews were conducted using an interview guide (see example in Supplementary Material B). Themes of the interviews included personal food behavior and relation to identity at work and at home, perception of sustainable diets, and changes made after the behavior change intervention (for second interview only). For example, at T2 participants were asked about their perception of the behavior change intervention and how they liked the information campaign. To investigate the effects of the behavior change intervention to behaviors in the home context, participants were asked if anything had changed since the last interview (T1), how the behavior change intervention influenced any behaviors at home or how they thought about sustainability and sustainable foods. By interviewing participants at two time points (pre- and post-intervention) and specifically questioning them about their experiences of the behavior change intervention, it was possible for us to draw inferences about the causative roots of any spillover effects that were discussed. The absence of a matched control condition within this study, however, means that such inferences are necessarily tentative.

During the semi-structured interview, participants were invited to complete a visual sorting task. The method was inspired by similar tasks used to assess individual environmental identity in the context of the Inclusion of Nature in Self scale (e.g., Schultz et al., 2004; Martin and Czellar, 2016). In the current context, the method was used to assess the relative centrality of three key terms (related to the behavioral intervention) to their self-identity. The first part of this task required participants to outline what they understood by the terms *environmentally-friendly-self*, *sustainability*, and *sustainable food*. The term *environmentally-friendly-self* was chosen to capture the essence of the participants' green identity. The term *sustainable food* was chosen as this mapped directly to the target of the behavior change intervention (i.e., encouraging more sustainable dietary choices). The term *sustainability* was chosen as it was thought to represent the more general concept driving the behavior change effort within the current study.

The second part of the task required interviewees to position the three aforementioned terms in relation to the outline drawing of a person (i.e., manikin) positioned within the center of a large piece of paper. They were asked to imagine that the manikin was a representation of themselves and to position each term (which had been printed separately on small pieces of paper) around the manikin based on the perceived centrality of the terms to them personally. For example, if a term was considered of central importance to the self, participants were instructed to place the term close to or overlapping the manikin. Conversely, if a term was considered of peripheral importance to the self, participants were instructed to place the term further away from the manikin.

The visual sorting task was carried out both pre- and post-intervention with a photograph taken of the arrangement reached by the participant after each session. By having participants complete the task twice, it was possible to learn more about the impact that the behavior change intervention had had upon the relative importance (i.e., centrality) of the aforementioned concepts to the participants' sense of self. Participants did not see the photograph of their responses to the pre-intervention sorting task before completing the post-intervention task.

## Findings

### Data Analysis Approach

All interviews were transcribed and then analyzed using thematic analysis (Braun and Clarke, 2006) via NVivo12. The analysis focused upon any references made to positive or negative spillover effects (or lack thereof) regarding ESBs following the behavior change intervention. We were particularly interested in any reported evidence of contextual spillover in ESBs from the workplace to home.

**Figure 3:**
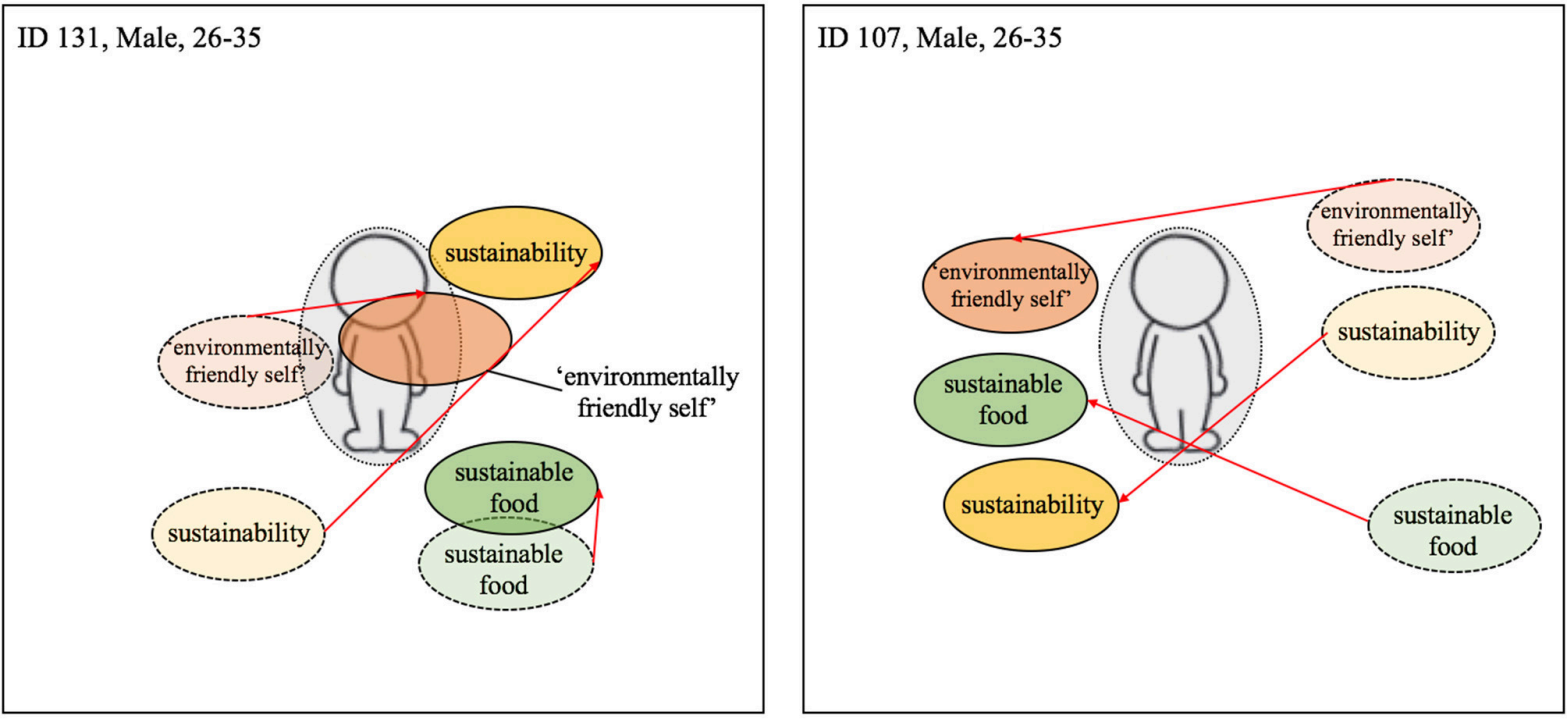
Visualization of centrality of identity—Reduced meat consumption. Dashed line = T1; solid line and darker coloring = T2; Red arrow indicates change from T1 to T2 (the colors were added after the analysis).

The thematic analysis of the interview transcripts, was supported by an analysis of participants' responses to the visualization task. This involved direct comparison of the placement of the three terms (i.e., *environmentally-friendly-self*, *sustainability*, and *sustainable food*) relative to the manikin pre- and post-intervention. The distance between the terms at each time point was analyzed by superimposing the photographs from the pre- and post-intervention sessions. The relative position of the terms and their distance from the manikin were visually inspected by looking at the extent of the shift of each term at T2 in comparison to T1. A gray circle encircling the manikin (see e.g., Figure 3; dashed line = T1; solid line and darker coloring = T2; red arrows indicate change from T1 to T2; the colors of the terms were added after the analysis for visualization purposes) was used to assist this process. Analysis of changes to the centrality of the terms focused on shifts in their relative distance from the center-point of the manikin only. Changes to the vertical or horizontal positioning of terms was not assessed (although it is acknowledged that horizontal or vertical axis movement using visual methods can be interpreted as a change, e.g., Meier and Robinson, 2004).

Reports of spillover derived from the interviews in combination with identified changes in the relative centrality of the key terms used within the visualization task were used to evaluate the theoretical framework proposed in this paper. It was hypothesized, for instance, that where there was evidence of a positive shift (centralization) in the centrality of the key terms used within the visualization task (indicative of successful integration into the self-concept) that this should be accompanied by verbal evidence of positive spillover effects. By contrast, evidence of a negative shift (decentralization) in the key terms would be indicative of a compartmentalization or emerging conflict of identity, which should be accompanied by verbal evidence of a lack of spillover or negative spillover effects.

### Spillover Effects and Change in Centrality

#### Positive Spillover

Evidence of positive contextual spillover (i.e., increase of ESBs similar or dissimilar to the target behavior of the intervention in the home context) was identified following the intervention. Specifically, some participants reported on a reduction of meat (or specifically red meat) consumption at home; an increase in consumption of British produce at home; and/or an increase in alternative small and “easy” positive changes to their lifestyles. There was also reported evidence of an increase in participants' awareness of the potentially negative environmental consequences of dietary choice following the intervention. To the extent that behavior change in the target context (workplace) was enforced (on account that all red meat was removed and white meat options were limited compared to normal), we feel that it is possible to infer that the increased tendency for people to shop for local, British produce at the supermarket (non-target context) can be taken as evidence of indirect spillover.

##### Reduction in meat consumption

A reduction in meat consumption (or specifically red meat consumption) at home after the behavior change intervention was identified as a dominant theme. The reported behaviors range from swapping red meat for chicken to an overall cut of meat consumption by trying a vegetarian month, a day a week meat free (e.g., meat free Monday) or generally eating less meat. For example, participant 131 reported a drastic reduction in meat consumption including meat free days, while 107 reported swapping red meat for white meat or generally trying to eat less meat.

“*we're trying to do the meat free Monday and that will then spillover to either the Tuesday or Wednesday cause we have got leftovers to eat as well” (131)*

“*yes, just replacing the majority of red meat with white meat and then moving over to some cos I mean generally we have meat at most meals and we can get away from that” (107)*

##### Increase in consumption of british produce

An increase in consumption of British produce was identified as another dominant theme. Reported changes in grocery shopping behavior included taking longer to make decisions and checking food labels. The dominant behavior change in supermarkets was the increase in buying local and British produce which participants reported either in addition to or instead of a reduction in meat consumption. Buying local food was often perceived as an easier alternative to reducing meat consumption or calculating the relative impacts of different product alternatives.

“*It is something I try and keep up with now a bit more instead of just giving it a lip service. […] a good example is I was shopping on Saturday and I went to get some strawberries. And there were like two different punnets [baskets]. […] The cheaper ones were from Spain whereas the other ones were from the UK. So, I thought, well I get the UK ones because we can grow strawberries, why do I need to get them from Spain. So little things like that, where the origin is in certain things, whereas previously I might not have” (131)*

“*[…] the only realistic thing that I could really keep tabs on it where my food is coming from. The other stuff like how much water is going into making it I don't even know how to work that out. […] I don't know how to choose in the supermarket whether something is grown under artificial conditions or whether it happens to be in season. […] just where the food comes from is an easily controllable thing where I can choose food by quite easily.” (106)*

##### Easy and small changes

Easy and small changes was identified as a third type of positive behavioral spillover in the interviews. Participants reported a variety of changes in ESBs at home which they described as being easier, more feasible or more controllable than reducing meat consumption. These changes included an increase in recycling behavior, consumption of smaller food portions, trying to reduce packaging, and using sustainable palm oil. Crucially, some participants saw these easier or smaller changes as being a step along the way to more substantial lifestyle change.

“*Ehm, the way I was perceiving it is just trying to look at small changes that can be made and it is looking at the bigger pictures, knowing red meat is worse than white meat which means just moving to more white meat instead of just red meats and that takes more steps of that ladder with less environmental impacts for foods.” (105)*

“*I suppose because the cost is high. […] taking up recycling is a bit of an extra pfaff but I can't really justify not doing it to myself. But changing you know how much meat and dairy I consume is, like it's a noticeable change. That is probably, it can be quite a painful change as well. I say painful but I'd miss it.” (117)*

##### Increased awareness

An increased awareness was identified as the predominant non-behavioral response to the workplace intervention. Participants reported that the sustainable food week had altered the way they thought about food (e.g., where and how it is sourced) and their diet. While this change in awareness was affiliated with positive contextual spillover among some (see above) other participants only reported on a change in their relative awareness or interest, without an associated change in behavior.

“*I don't really know there has been any other kind of behavioural changes. It is more like just thinking. The way I think has definitely changed” (104)*

“*It wasn't so strong that I wanted to go and do extra research on it. But it was enough to just make me aware, I suppose” (102)*

#### Centrality of Identity and Positive Spillover

Where verbal evidence of positive spillover had been reported by participants, we also looked for any relative change in the centrality of the core terms used within the visualization task.

##### A reduction in meat consumption

Participants that reported a reduction in meat consumption following the workplace intervention were found to position the three terms closer to the manikin (i.e., the self) in the post-intervention task relative to the pre-intervention task (see Figure 3). For example, participant 131, who reported consuming less meat at home following the intervention, positioned the term *environmentally-friendly-self* more centrally on the manikin at T2. Similarly, participant 107, who reported a change from red to white meat consumption post-intervention, also positioned all three terms closer to manikin at T2. We argue that the relative overlap with the manikin itself can be taken as a register of the extent (full/partial) of the integration of the terms into the self.

##### An increase in consumption of british produce

**Figure 4:**
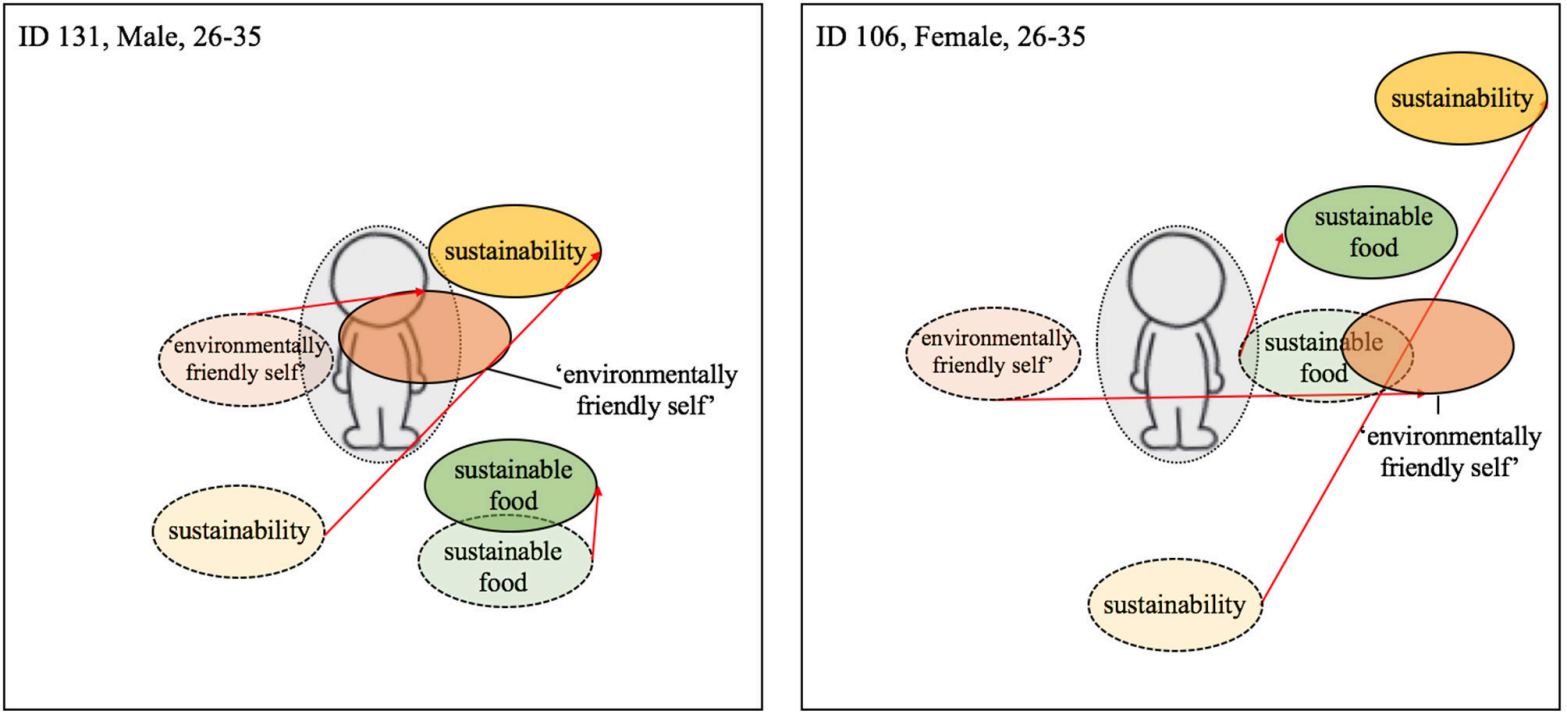
Visualization centrality of identity—Local food; Dashed line = T1; solid line and darker coloring = T2; Red arrow indicates change from T1 to T2 (the colors were added after the analysis).

**Figure 5:**
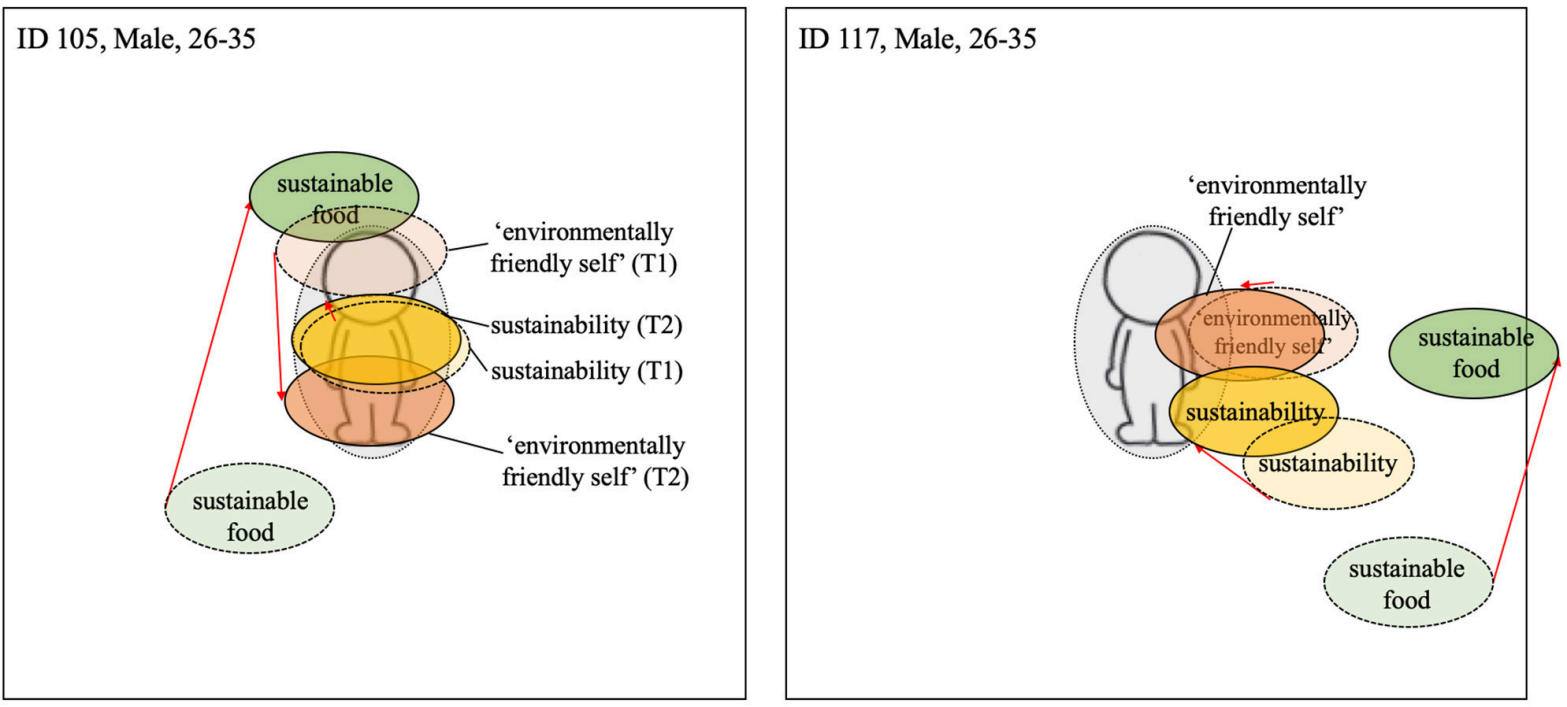
Visualization for centrality of identity—“Easy”and small changes; Dashed line = T1; solid line and darker coloring = T2; Red arrow indicates change from T1 to T2 (the colors were added after the analysis).

While some participants reduced both their meat consumption and increased their consumption of British produce, others only increased their consumption of local produce at home. For former group, the centrality of all terms typically increased in centrality post-intervention. For example, participant 131 positioned all three terms closer to the manikin at Time 2 (see Figure 4). For the individuals that only increased their consumption of British produce, the shift in centrality was much less apparent or a slight outward movement of some terms occurred, e.g., for participant 106 the term *sustainable food* (see Figure 4). This sign of non-integration of some terms and simultaneous integration of others could indicate a compartmentalization of sustainability and environmentally-friendly-self from sustainable food. This compartmentalization allows the individual, potentially guided by the consistency principle, to continue meat consumption while perceiving themselves as more pro-environmental. A similar shift pattern can be seen with participant 117 (Figure 5) and participant 126 (Figure 7), both of whom did not report upon changes their meat consumption behavior at home.

##### Easy and small changes

Among both participants reporting engaging in easy and small behavioral changes in response to the workplace intervention, positioning of all three terms became more centralized. As can be seen in Figure 5, the terms *sustainability* and *environmentally-friendly-self*, although relatively central pre-intervention, became more centralized post-intervention. The term *sustainable food* also became more central, but remained rather peripheral to the other terms. Moreover, the movement and centrality of the term *sustainable food* was less evident than among those participants showing a post-intervention shift in meat consumption at home (see Figures 2, 4).

##### Increased awareness only

**Figure 6:**
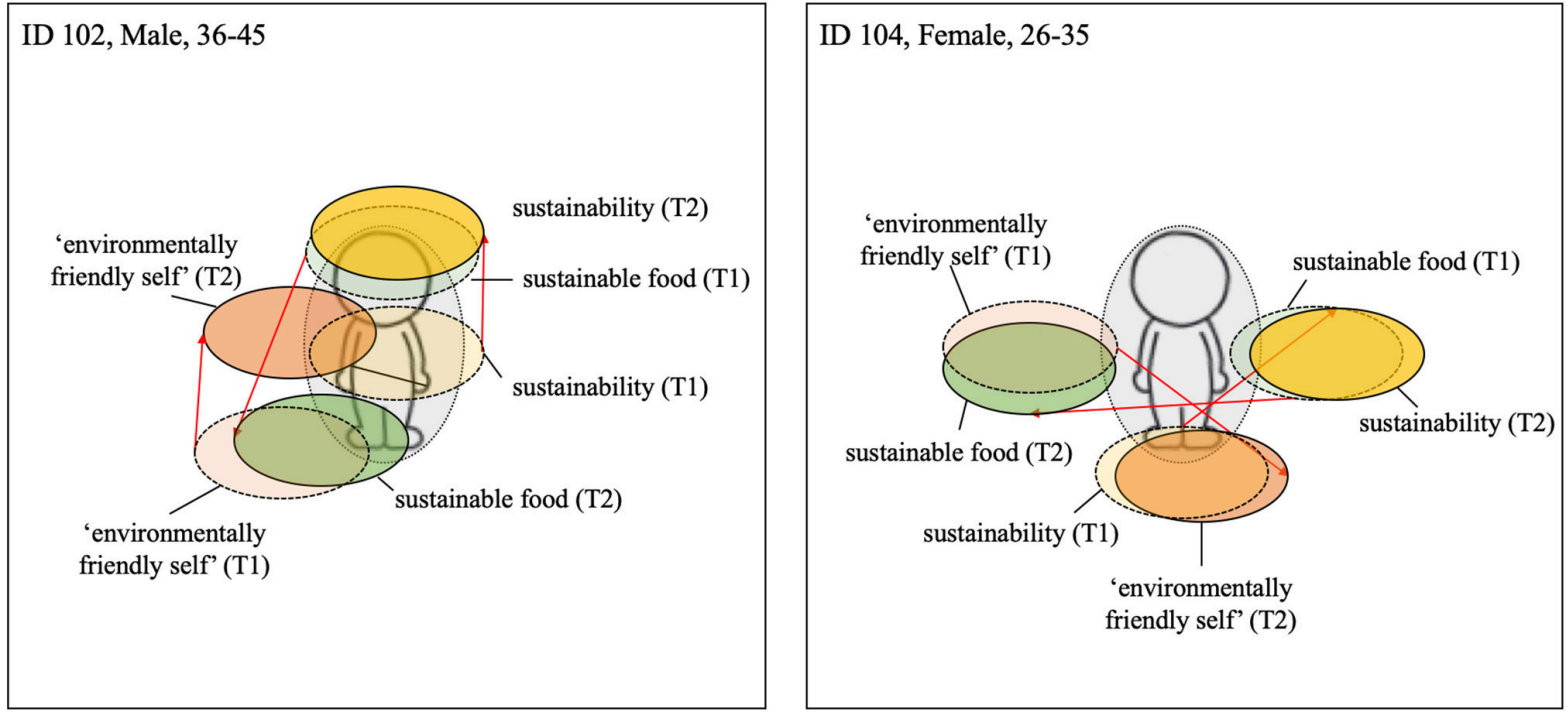
Visualization of centrality of identity—Increased awareness and concern; Dashed line = T1; solid line and darker coloring = T2; Red arrow indicates change from T1 to T2 (the colors were added after the analysis).

Participants reporting only an increased awareness of the implications of dietary choice post-intervention showed no change in centrality of the various terms. Similar to those individuals reporting evidence of spillover effects, however, all three terms were placed relatively centrally on the manikin. For example, participants 102 and 104 arranged all three terms relatively close to the manikin both before and after the intervention but any changes in centrality between these two time-points were marginal (see Figure 6).

#### Lack of Spillover

A lack of spillover was identified as an existing but less prominent theme. Where a lack of spillover was reported, this tended to be accompanied by excuses and justifications of why household behavior change did not occur.

##### Reaffirmation and mental accounting

Participants would often praise themselves for the ESBs they already engaged in so as to undermine the need for further change. Similarly, participants reported upon engaging in compensatory actions regarding their meat consumption so as to excuse themselves from changing this behavior. For example, participant 105 apparently protected their meat consumption habits by talking about the carbon emissions saved by buying their beef locally rather than from overseas.

“*British beef is going to have less CO*_2_ *emissions involved than getting beef from New Zealand. So, it doesn't have to be flown half way around the world to get here. So, it's always looking at carbon offsetting, there is always ways of looking at reducing CO*_2_ *emissions in other ways as well. […] And something that has always been a big thing for me is making sure that it's British produced, regardless of what I'm eating” (105)*

##### Conditional intention to change behavior

An intention to change dietary choices at home if certain pre-conditions were met (e.g., there was no extra effort and/or cost associated with doing so) was identified as another dominant pattern. Participants speaking about their intentions to change would often use the future tense and/or hypothetical scenarios to describe the likely future behaviors, but did not report upon having made any actual changes to the diets as a result of the workplace intervention.

“*I would, it is something that I would consider kind of maybe doing like one or two days a week having like a conscious you know what, I'm going to eat vegetarian for a couple of days a week. And try vegetarian food. But it is not something I would. It would have to be an easy thing to do.” (110)*

#### Centrality of Identity and a Lack of Spillover

**Figure 7:**
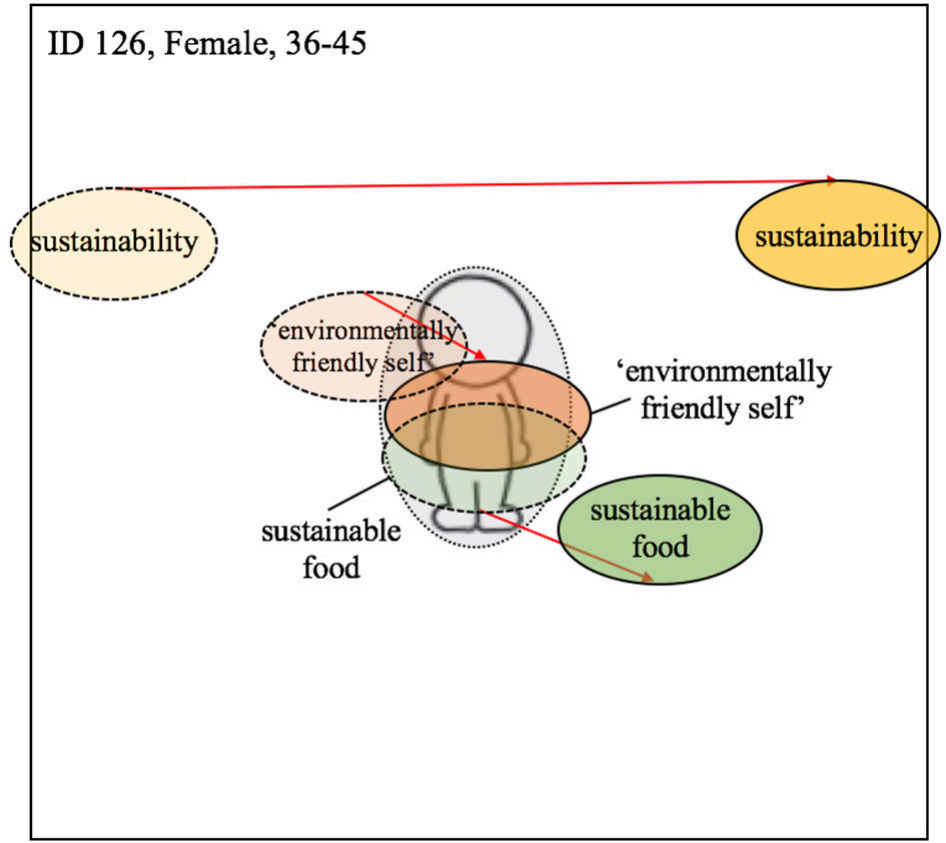
Visualization of centrality of identity—Lack of spillover; Dashed line = T1; solid line and darker coloring = T2; Red arrow indicates change from T1 to T2 (the colors were added after the analysis).

Among those evidencing an apparent lack of spillover, the results of the visualization task presented a mixed picture. While all three terms became slightly more central for some participants, for others some of the terms increased in centrality while others decreased in their centrality (see Figure 7, ID 126). For example, for participant 126 the centrality of *sustainable food* decreased, while the term *environmentally-friendly-self* became more central and the term *sustainability* did not change noticeably.

**Figure 8:**
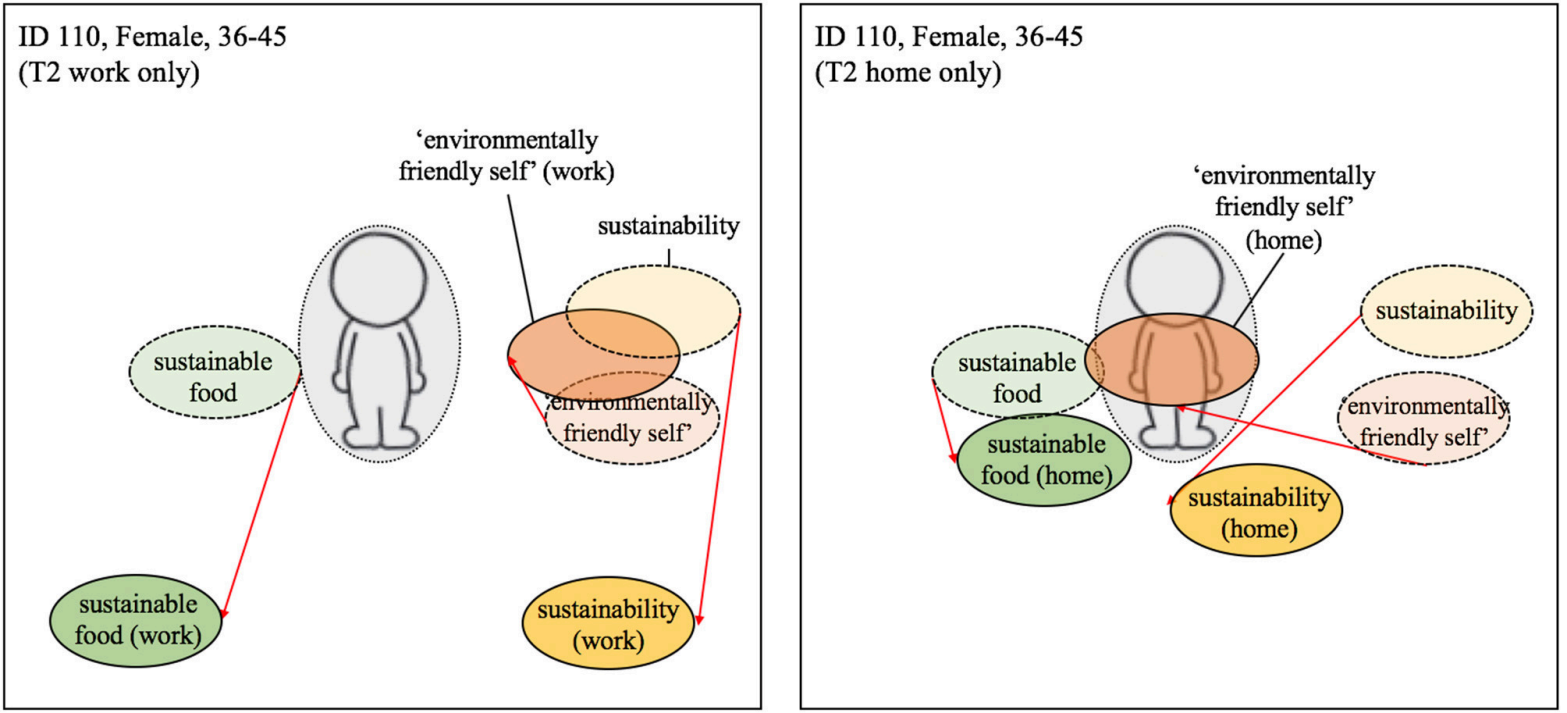
Visualization of centrality of identity—Compartmentalization of work and home. Dashed line = T1; solid line and darker coloring = T2; Red arrow indicates change from T1 to T2 (the colors were added after the analysis). Participant differentiated between self at work and home at T2.

Two participants showed explicit compartmentalization of the terms, positioning the terms differently for the home and work context (see Figure 8; ID 110). For example, for participant 110 the term *environmentally-friendly-self* became very central post-intervention in the home context but only slightly so in the workplace. While the terms *sustainability* and *sustainable food* were found to increase slightly in centrality in the home but decrease in centrality within the workplace setting.

#### Negative Spillover

There was little evidence of negative spillover among our interviewed sample, although anecdotally there were reports of negative behavioral responses to the intervention (e.g., some employees went to a shop nearby to by meat and came back to add the meat to the vegetarian sandwiches). One interviewee (participant 129) did, however, describe a response to the sustainable food week that could be taken as bordering upon negative spillover. Specifically, while not reporting on an increase in negative environmental behaviors following the workplace intervention *per se*, participant 129 did respond negatively to the “meat-reduction” theme of the intervention; verbalizing resistance to its aims and cynicism about its benefits.

“*I don't think people would stop eating meat. And I think it would have far more disastrous consequences in terms of people's health, in terms of economies, things like that, if people stopped eating meat. […] It was just a bit biased, the questions were a bit, you couldn't answer anything other (laughing) oh my, we should all be eating this sustainable food.” (129)*

#### Centrality of Identity and Negative Spillover

**Figure 9:**
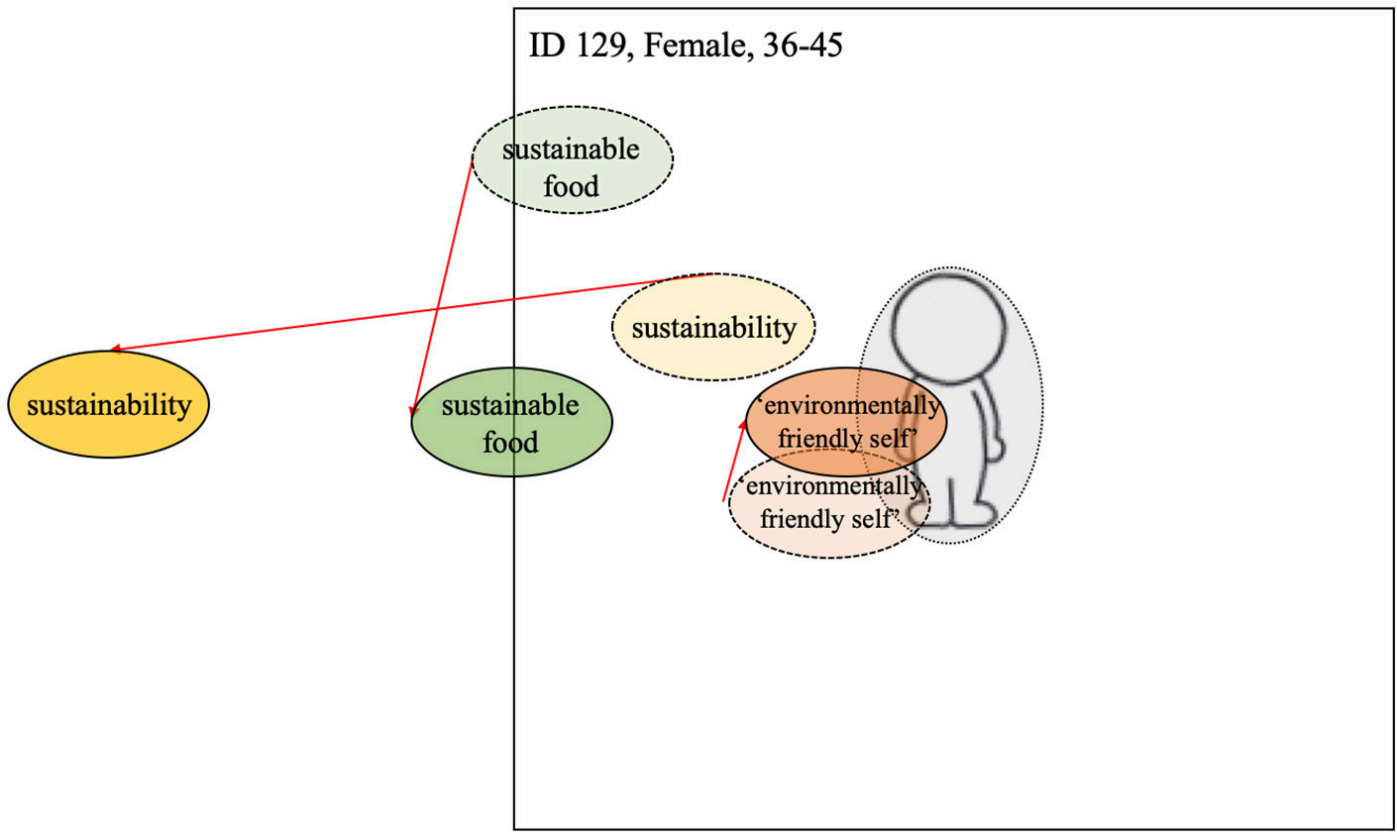
Visualization of centrality of identity—Negative spillover. Dashed line = T1; solid line and darker coloring = T2; Red arrow indicates change from T1 to T2 (the colors were added after the analysis).

The negative reactions aired by participant 129 were accompanied by interesting changes to the centrality of the three terms within the manikin task. While the term *environmentally-friendly-self* became slightly more central, the term *sustainable food* changed only marginally, and the term *sustainability* decreased considerably in centrality (see Figure 9). In fact, the term *sustainability* was decentralized from a position relatively close to the manikin (pre-intervention) to being off the paper (post-intervention).

## Discussion

The current article proposed a theoretical framework, based upon the principles of identity process theory (IPT), designed to help explain the emergence of positive and negative spillover effects (and absence thereof) in relation to ESBs. In addition to outlining a theoretical framework, we provide empirical evidence in support of the framework from a real-life behavior change intervention in a workplace environment. Below, we discuss the results in light of the theoretical framework and identify limitations and gaps that future research should address.

### Evidence for Theoretical Framework

#### Positive Spillover

Encouragingly, our study provides evidence for direct, positive contextual spillover from the workplace to the home setting (i.e., an increase in ESBs similar to the target behavior of the intervention in the home context), as well as more indirect spillover between behaviors across contexts (i.e., an increase in ESBs dissimilar to the target behavior of the intervention in the home context). Specifically, evidence for *direct* spillover effects in the form of decreased meat consumption at home was identified among our participants who participated in the pre- post-intervention study. Importantly, this evidence of contextual spillover was accompanied by a clear and associated increase in the centrality of a number of terms thought to map to a person's green identity (i.e., *environmentally-friendly-self*, *sustainability*, and *sustainable food*). We argue that, consistent with our theoretical framework, these initial findings are indicative of individuals having successfully (i.e., fully) integrated the tenets of the workplace intervention into their self-concept. In turn, we feel that this integration prompted a greater desire among these individuals to act pro-environmentally (yielding the recounted spillover effects) due to a strengthening of green identity and a desire to act consistently and in accordance with this identity (guided by the *consistency principle*).

The findings perhaps point to the nature of the integration that occurred within our respondents. Specifically, where there was clear movement in the centrality of the terms within the visualization task, one could infer evidence of accommodation. That is, the obvious changes to the centrality of the terms could be taken to illustrate change within the identity structures of the respondent. By contrast, where less obvious movement was in evidence, one might infer there was a strengthening of existing identity via assimilative processes. These conclusions are speculative, however, and do require further investigation in future research. This is particularly important being that one could also infer that an increase in the centrality of all the terms might be a simple by-product of a strengthening of existing identity as opposed to being illustrative of an adaptation to identity *per se*.

Where *direct* contextual spillover did not occur (i.e., in terms of meat consumption at home and work), there was still evidence of more *indirect* positive spillover effects toward certain similar (e.g., increased selection of domestic produce) and dissimilar behaviors (e.g., increased recycling) in home-relevant contexts (including in the household and while shopping for produce to use at home). This *indirect* positive spillover was still associated with the increased centrality of the terms, although to a lesser extent than in the case of direct contextual spillover. We argue that this can again be seen as evidence of a strengthening of green identity in response to the tenets of the workplace appeal, but in the face of conflict driven by a person's desire to continue to eat meat. Crucially, however, participants still sought to act in accordance with their strengthened sense of green identity by actively engaging in alternative but ostensibly easier, more controllable and/or more personally desirable acts.

While indirect positive spillover is clearly desirable, we argue that an absence of change in the target behavior of an appeal across contexts could be evidence of partial or incomplete assimilation of the tenets of that appeal into ones self-identity. That is, while there is a general strengthening of one's green identity in response to the appeal (which drives people to wish to act more pro-environmentally), there is failure to fully integrate and respond to the more specific tenets of the appeal. In turn, we argue that indirect positive contextual spillover is therefore a product of a general increase in a person's desire to be pro-environmental but in the face of dispositional resistance to cross-contextual change in the specific target behavior. That being said, indirect positive spillover could also be expected to occur in response to full integration of the tenets of a persuasive appeal but in a situation where there are perceptively situational barriers to enacting direct cross-contextual spillover (e.g., due to satisfying the wishes of others at meal times). We feel that future research could usefully explore the extent to which indirect positive spillover is: (a) a product of full and/or partial identity-integration; and (b) mediated by dispositional resistance or perceived situational constraints.

#### Evidence for a Lack of Spillover

Unlike positive spillover, lack of spillover was typified by mixed changes in centrality of terms used within the visualization task. We argue that this is indicative of a relative failure of the intervention to produce enduring and substantive change to a given participants' green identity.

In line with our theoretical model, for instance, there was evidence of one participant seemingly resolving the conflict posed by the intervention by contextually separating (i.e., fully compartmentalizing) their workplace and home-life identities (see participant 110; Figure 8). Importantly, there was also some evidence of the other conflict management strategies predicted by our model, in particular *suppression*. However, as opposed to the suppression of green identity relative to other identity characteristics *per se*, the suppression appeared to relate to the relative importance of dietary choice *within* one's green identity. For example, participant 126 showed a post-intervention increase in the centrality of *environmentally-friendly-self* combined with a decrease in the relative centrality of the term *sustainable food*. We argue that this is again illustrative of the conflict that arose in the participant following the behavior change intervention (i.e., a growing awareness of the need to be pro-environmental but a desire to continue eating meat). Rather than choosing to proactively adapt their behavior, however, they ostensibly resolved the conflict by more clearly distinguishing dietary choice (peripheral) from their strengthened desire to be more environmentally friendly (more central; see Figure 9). In diminishing the relative centrality of dietary choice to green identity in this way, the participant could then more easily maintain a perception of themselves as pro-environmental while licensing their continued desire to eat meat.

Interestingly, in the context of a lack of spillover, this resolution appeared to be retrospectively justified, with people drawing upon past pro-environmental actions in order to license the lack of change in dietary behaviors. This meant there was no observable direct or indirect contextual positive spillover. Such retrospective justification in relation to ESBs has been identified in other research (e.g., compensatory green beliefs, Hope et al., 2018) and is apparently motivated by an *extrinsically* motivated desire for social approval.

The failure of the intervention to evoke substantive change in all respondents is perhaps to be expected. There is some evidence to suggest that green identity stems from relatively enduring characteristics like a person's biophilic tendencies (Hinds and Sparks, 2009; Fleury-Bahi et al., 2017). To the extent that the basis of one's green identity is derived from such enduring constructs, one might only anticipate a one-off behavior change intervention (like ours) to evoke registerable change among those with stronger biophilic tendencies. This is not to say that such change would not be evoked among less biophilic individuals under different conditions (e.g., in response to a more sustained, longitudinal intervention); however, in the context of the current study, such individual differences might have had more of an impact. Again, this conclusion is speculative at the current time and warrants further investigation within future work.

#### Evidence for Negative Spillover

According to our preferred definitions of spillover as relating to observable behavior change in response to an intervention, we recorded no categorical evidence of negative spillover effects within our study. That said, we did receive anecdotal evidence of negative spillover effects occurring among employees of the host company. The sentiment underlying these negative effects was, however, captured by one of our interviewees (participant 129; Figure 8) who, while not reporting to have personally sabotaged the campaign or engaged in negative environmental acts, illustrated a clear resistance to the intervention. It was this negative reactance that distinguished participant 129 from those participants evidencing a more benign lack of spillover (e.g., participants 110 and 126; Figures 8, 7).

While care must be taken in drawing firm conclusions about the mechanisms underpinning negative spillover from this one case (although the power of single cases should not be underestimated, see Eisenhardt and Graebner, 2007), there are some indicators within the participant's responses that speak to aspects of our theoretical model. For example, their verbal resistance to the campaign during the interview was partnered by a decentralization of two of the terms *sustainability* and *sustainable food* within the visualization task. And in the case of *sustainable food* this decentralization was so extreme so as to effectively remove the term from the table.

We argue that this finding could be taken as evidence of the intervention having threatened an important part of the participants' self-image (e.g., Giner-Sorolila and Chaiken, 1997), thus stimulating the emergence of conflicting identities (i.e., meat eater vs. sustainable person). It is possible that the participant sought to resolve this conflict by figuratively removing the discussion of dietary choice (and reduction in meat consumption) from wider discussions about the need to be more environmentally sustainable. What perhaps prevented the negative sentiment evolving into actual negative behavioral spillover in this case was the countermovement of the term *environmentally-friendly-self* toward a position of greater centrality. Crucially, the trends reported here were only identified in one of the 13 participants and whether or not the increased centrality of the term *environmentally-friendly-self* was indicative of an *internal* strengthening of the participant's green identity or an *extrinsic* response to the interview context (i.e., a desire not to appear un-environmental in from of the interviewer), remains open. As such, these explanations need further investigation in future work.

#### Implications, Limitations, and Future Directions

The principal aims of the current study were to propose and provide some initial supporting evidence for a theoretically-informed, conceptual framework for understanding contextual (and broader) spillover. Our initial findings from post-intervention, qualitative interviews in association with the visualization task would appear to be broadly consistent with the predictions made within the conceptual framework and thus point to identity—and the relative success with which the tenets of behavior change appeals are integrated into one's identity—as being a mediator of the likelihood that contextual spillover will occur.

On the basis of our exploratory research, it would appear that where the tenets of an appeal are fully integrated (i.e., adapted), then there is an increased likelihood of observing *direct* positive contextual spillover (i.e., people taking the theme of a behavior change intervention from one context and transferring it to other contexts). Conversely, our initial findings show less successful integration is liable to lead to more *indirect* positive contextual spillover effects (i.e., people altering behaviors other than that target behavior), a lack of spillover or even (in some cases) negative spillover. That said, we did not find firm evidence of negative contextual spillover within our study.

While there are certain limitations to this research (outlined below), we argue that the initial findings hold a number of real-world implications also. Chiefly, by firmly implicating identity threat as a limiter of positive contextual spillover, we feel that behavior change interventions targeting change in ESBs, should be accompanied by efforts to reduce the potential threat felt by recipients. There is already growing evidence of the value of priming pride (as opposed to guilt) as a means of encouraging people to engage in more ESBs (e.g., Bissing-Olson et al., 2016) which is consistent with this suggestion. However, we would also argue that another option could be to draw upon the principles of self-affirmation theory (Sherman and Cohen, 2006). The principles of self-affirmation have already been used successfully in the domain of health behaviors. Studies show that by having people bolster their perception of self-worth before receiving ostensibly threatening (e.g., health risk) information, decreases defensive processing and is a good means of increasing the likelihood they will respond appropriately.

Care should, though, be taken in generalizing from the findings of this study due to a number of limitations. Aside from the obvious limitations to the transferability of the study posed by the small sample and the fact that this study was based upon a one-off intervention, with a narrow focus on meat consumption and confined to one particular workplace environment, there are other theoretical and methodological limitations to bear in mind.

Theoretically, for example, our model focuses solely on the role that identity processes might play in explaining contextual spillover. While this decision was made on the basis of the recognized importance that identity has in guiding behavior (Van der Werff et al., 2014), other psychological variables—such as environmental attitudes (e.g., De Dominicis et al., 2017), environmental values (e.g., Steg et al., 2014), or social norms (Keizer and Schultz, 2018)—are also known to shape people's ESBs. As such, these variables might also be anticipated to play a role in helping to explain the mechanisms behind the emergence of contextual spillover effects. Beyond dispositional characteristics, there are also certain situational characteristics that we did not consider within the current study but which could affect the likelihood of contextual spillover, e.g., the perceived similarities and differences in the intervention and spillover contexts (Littleford et al., 2014).

As such, we argue that future research could usefully seek to expand upon our proposed theoretical framework in order to recognize more of the potential psychological and situational factors governing contextual spillover. Such research might, for example, seek to delve deeper into the factors accounting for the emergence of indirect spillover in the absence of direct spillover (e.g., studying the role of compensatory beliefs and behaviors in inhibiting direct spillover effects, see Hope et al., 2018); or investigate how social dynamics affect the likelihood of contextual spillover occurring within group settings (e.g., looking at how the opinions or actions of others promote or inhibit spillover within and between social contexts, Sinclair et al., 2012).

Methodologically, our visualization task, while based upon existing research (e.g., Martin and Czellar, 2016), is a novel approach in assessing changes in the centrality of identity elements, particularly in the context of spillover. Further research should be conducted to further validate the use of this approach. Such validation might, employ “think aloud” methodology; where people can privately talk through their decisions regarding the positioning of the terms, out of the face-to-face presence of the experimenter (Kaklamanou et al., 2013; Hope et al., 2018) or in the form of a think aloud-visualization task, where participants could talk through their decisions regarding the positioning of the terms in an open manner. Not only would such studies likely yield a verbal account of the reasoning behind placement decisions (e.g., the extent to which the changes reflect a conflict in a participants identity) but also the reduction in the immediacy of the experimenter which could be introduced using such methods (vs. an interview) would likely yield less demand artifact, socially desirable responding or other experimenter induced bias (e.g., the Pygmalion effect).

Furthermore, in terms of methodology, we recognize that the absence of control condition within the current study means that any claims of cauzation within the findings are necessarily tentative. We argue that more tightly controlled study designs, such as the use of multilevel experiments (e.g., Sinclair et al., 2012), could now be used to help confirm the assumptions inferred within this study, and other, spillover research.

Finally, we feel that in the future it would be prudent to include other key terms in the visualization task in order to explicitly test some of the assumptions arising from the current study. In particular, including terms that might be seen to represent conflicting identities that might form in relation to dietary choice could be interesting. For instance, the inclusion of the term *meat-eater* within the current study could have helped to provide more direct evidence of the conflict (or conflicting identities) that had been stimulated by our interventions. Also, to the extent that some people might question the health risks and/or benefits of eating animal-protein, introducing terms like *healthy eater* could also prove interesting in this regard. A further option could be to work with participants directly to identify pairs of terms relating to dietary choice, identity and environmental sustainability that they find to be opposing (similar to Q-sort methodology; Brown, 1996). In doing so, one could not only identify the terms that are subjectively important to each respondent (providing a clearer picture of their specific “consumer” identity) but one could then investigate how these opposing terms shift in relation to one another in response to a behavior-change intervention.

## Conclusion

This paper used identity process theory as the basis for introducing a theoretically informed framework for behavioral spillover. Our focus on contextual spillover effects (workplace to home) was designed to directly address a current hole in the literature; however, we feel that the framework we have created should also be directly applicable to understanding other forms of behavioral spillover also. Results of an explorative visualization task and interview method provided initial evidence of direct and indirect positive contextual spillover effects, with comparatively less evidence of a lack of spillover and a relative absence of reported negative spillover. Consistent with the conceptual model developed within this study, whether or not positive spillover was observed seemed to be tied to the extent to which the tenets of the behavior change appeal (in this case designed to reduce meat consumption) were integrated into a person's sense of self. Future research is now required to test and evaluate the theoretical framework and confirm its relevance for understanding spillover effects, validate the methodological approach used in this initial study and address some of incumbent limitations.

## Ethics Statement

Ethics approval for this research has been granted by the University of Sheffield Management School Ethics Committee.

## Author Contributions

The research outlined in this paper constitutes part of CV's Ph.D. thesis. CV was involved in developing the research question, designing the study, collecting and analyzing the data, and writing the manuscript. CJ was involved in co-writing all sections of this manuscript. CJ, DG-S, and CO were involved in developing the research questions and methodological approach used within this research, as well as editing the manuscript.

### Conflict of Interest Statement

The authors declare that the research was conducted in the absence of any commercial or financial relationships that could be construed as a potential conflict of interest.
