# Supplementary material for: Understanding Contextual Spillover: Using Identity Process Theory as a Lens for Analyzing Behavioral Responses to a Workplace Dietary Choice Intervention
Source: Front Psychol. 2019 Mar 1;10:345. doi: 10.3389/fpsyg.2019.00345 (PMC6405690; doi:10.3389/fpsyg.2019.00345)
Supplement: Supplementary file 1 [file Table_1.DOCX]

Supplementary Material A

# Stages of change scale and distribution among interviewees

*This scale was adapted from a scale developed by Bamberg (2013).*

Which of the following statements best describes how you feel about your current level of sustainable food consumption and whether you have any plans to try to change it? (Please choose which statement fits best to your current situation)

Note: Sustainable food is here understood as adopting a diet with lower environmental impact. Amongst other things this can include a preference for organic, local, and seasonal food and reduced meat and dairy consumption.

- [STAGE 1] At the moment, I don't pay attention to whether my food is sustainable or not. I'm happy with the current food I consume and see no reason why I should change it.
- [STAGE 2] At the moment, I still don't eat sustainable food for most of my meals. I would like to increase my sustainable food consumption, but, at the moment, I feel it would be impossible for me to do so.
- [STAGE 3] At the moment, I don't eat sustainable food for most of my meals. I'm currently thinking about changing some or all of these meals to be more sustainable, but at the moment I'm unsure how I can do this, or when I should do so.
- [STAGE 4] At the moment, I don’t eat sustainable food for most of my meals, but it is my aim to increase my current level of sustainable food consumption. I already know which meals I will replace and which alternative foodstuff I will use, but, as yet, have not actually put this into practice.
- [STAGE 5] Because I'm aware of many problems associated with unsustainable food consumption, I already try to use sustainable food alternatives as much as possible. I will maintain or even increase my already high level of sustainable food consumption in the next months.
- [STAGE 6] As I already eat sustainable food all the time, increasing my level of sustainable consumption is not currently an issue for me.

| **ID** | **Gender** | **Age** | **Stage of change at T1 (based on survey)** |
| --- | --- | --- | --- |
| 104 | Female | 18-25 | STAGE 2  At the moment, I still don't eat sustainable food for most of my meals. I would like to increase my sustainable food consumption, but, at the moment, I feel it would be impossible for me to do so. |
| 108 | Female | 26-35 | STAGE 3  At the moment, I don't eat sustainable food for most of my meals. I'm currently thinking about changing some or all of these meals to be more sustainable, but at the moment I'm unsure how I can do this, or when I should do so. |
| 117 | Male | 26-35 | STAGE 3 |
| 107 | Male | 26-35 | STAGE 3 |
| 110 | Female | 36-45 | STAGE 3 |
| 106 | Female | 26-35 | STAGE 3 |
| 129 | Female | 36-45 | STAGE 3 |
| 112 | Female | 26-35 | STAGE 4  At the moment, I don’t eat sustainable food for most of my meals, but it is my aim to increase my current level of sustainable food consumption. I already know which meals I will replace and which alternative foodstuff I will use, but, as yet, have not actually put this into practice. |
| 105 | Male | 26-35 | STAGE 4 |
| 126 | Female | 36-45 | STAGE 5  Because I'm aware of many problems associated with unsustainable food consumption, I already try to use sustainable food alternatives as much as possible. I will maintain or even increase my already high level of sustainable food consumption in the next months. |
| 131 | Male | 26-35 | STAGE 5 |
| 102 | Male | 36-45 | STAGE 5 |
| 132 | Male | 46-55 | STAGE 6  As I already eat sustainable food all the time, increasing my level of sustainable consumption is not currently an issue for me. |
