# Supplementary material for: Understanding Contextual Spillover: Using Identity Process Theory as a Lens for Analyzing Behavioral Responses to a Workplace Dietary Choice Intervention
Source: Front Psychol. 2019 Mar 1;10:345. doi: 10.3389/fpsyg.2019.00345 (PMC6405690; doi:10.3389/fpsyg.2019.00345)
Supplement: Supplementary file 2 [file Table_2.DOCX]

Supplementary Material B

# Interview guide for interviews at T2 (after the behaviour change intervention)

- What did you think about the sustainable food week?
- What did you think about the information on the impact of food on the environment (provided on the table talkers)
- How did you react to the information?
- How did it make you feel?
- How did it make you think about your food consumption?
- How did it make you think about yourself?
- What did you think about the new food choices?
- How did you react when you found the new food choices in the canteen?
- How did you like having less meat options?
- How did it make you feel?
- How did your colleagues react about the changes we made?

Now I would like to reflect with you how your perception and behaviour around sustainability and food is since we last met. I will ask you a few things I asked you last time already.

- What does sustainability mean to you?
- What role does it play in your everyday life? [Can you give examples?]
- What does sustainable food mean to you?
- What role does sustainable food play for your own food consumption? [Can you give examples?]

[Visual Sorting Task: Place manikin in front of participant and provide the three terms, if possible in no specific relation to the manikin].

- What do you understand under these terms?
- Imagine that is your Self (pointing at the manikin). Could you sort these words around? Close to you means it is quite central to you, far away means it is not very central to you.
- What role does sustainability play for who you are as a person?
- How has it changed since we last spoke?
- What role does sustainable food play for who you are as a person?
- How has it changed since we last spoke?
- How did the changes we made in the canteen influence what you eat at home, if at all? [Can you give examples?]
- How do you think about sustainable behaviours at home since we last spoke?
- How did the sustainable food week make you think about other sustainable behaviours?
- How do you feel about yourself when you act sustainably at work?

Thank you for your time. Do you have any other comments/questions/feedback
