# Supplementary material for: Understanding Contextual Spillover: Using Identity Process Theory as a Lens for Analyzing Behavioral Responses to a Workplace Dietary Choice Intervention
Source: Front Psychol. 2019 Mar 1;10:345. doi: 10.3389/fpsyg.2019.00345 (PMC6405690; doi:10.3389/fpsyg.2019.00345)
Supplement: Supplementary file 3 [file Data_Sheet_1.PDF]

Assessment of food recommendations for behaviour change intervention

Recommendation based on WWF Livewell Report and/or FCRN	Environmental Impact	Feasibility (behaviour plasticity)	Measurability of behaviour
Diversity – a wide variety of foods eaten.[1,2]	MEDIUM	HIGH	MEDIUM
Moderate meat consumption. [1,2,] (Replace meat with peas, beans and pulses, tofu, nuts, and other plant sources of protein.)	HIGH	HIGH	HIGH
Increase of plant based foods[1,2,] (minimally processed tubers and whole grains; legumes; fruits and vegetables)	HIGH	HIGH	HIGH
Moderate milk and dairy products included in diet but seek out plant based alternatives[2]	MEDIUM	HIGH	HIGH
Reduce food waste[1]	MEDIUM	HIGH	MEDIUM
Eat fewer foods high in fat, sugar and salt[1,2]	LOW	MEDIUM	MEDIUM
Certified fish and other foods from sustainable sources[1,2] (small quantities of fish)	MEDIUM	MEDIUM (often dependent on income and transparency/ awareness of labels)	MEDIUM
Tap water in preference to other beverages[2]		MEDIUM (dependent on taste and availability of fresh water. In the UK normally high)	MEDIUM
Balance achieved between energy intake and energy needs[2]	MEDIUM	HIGH	LOW
Oils and fats with a beneficial Omega 3:6 ratio such as rapeseed and olive oil[2]	LOW	MEDIUM	LOW

Based on assessment presented above, a reduction in meat consumption and increase in fruit and vegetable consumption was chosen as target behaviours for the behaviour change intervention. Sources:[1]WWF Livewell Report 2011, [2]FCRN

Employee survey

We asked 157 employees in Sheffield about sustainable food in the canteen

Top 5 sustainable food suggestions for the canteen:

LOCAL FOOD

“Locally produced Meats, Vegetable and fruit”

“More hot, freshly cooked vegetables and locally grown fruit”

“Local meat and free range”

”Local meat/ dairy produce”

“On the Free Salad Bar, get rid of that horrible processed Beef/Turkey and Ham and put proper, locally sourced meat there, instead”

VEGAN & VEGERATIAN OPTION

“more vegetables, more meat free options, sandwich fillers, options for salads (e.g. feta cheese, couscous, pesto, Quorn)”

“Healthy vegetarian options that are NEVER there”

“I don't eat at times due to no proper veg options available”

“Veggie options that don’t use Quorn”

“More veggie options for meat alternatives”

“Some warm vegetarian options, vegan and fat free options, non gluten products also”

SEASONAL FOOD

“Seasonal foods on the salad bar”

“season food used more i.e. in winter have casseroles and in summer go for more stir fry options”

“local, seasonal vegetables and fruit”

“vegetables that are in season”

FRESH FOOD

“Fresh food would be better, rather than processed food to cater for the hundreds that eat there.”

“Fresher meat instead of processed.”

“fresher salad”

FOOD SOURCE

“Meat and fish from sustainable/environment ally friendly sources”

“free range eggs, free range meat, fish that advises of its origin + if line caught”

“free range eggs + meat”

“More thought into sourcing food other than 'what can we buy in bulk cheaply”

Source: Selected quotes from the survey T1

Behaviour change intervention week 10th-14th July

WHAT DID WE CHANGE?

- Reduction of available meat options from 8-10 to 1-3
- Information material was provided on tables

INFORMATION MATERIAL

The information material was developed for the sustainable food week. Each day, different material was provided in the form of 'table talkers' that were placed on each table in the canteen.
The themes were CO_2 emissions, water consumption, and resources (space, energy) needed to produce foodstuff, particularly meat.

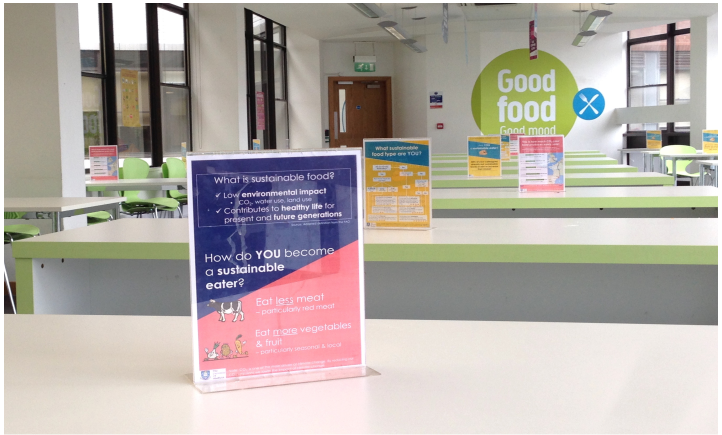

Above: Information provided during sustainable food week.

For 1 week (10th-14th July) we changed the food in the canteen and implemented an information campaign along side.

Food changes: The food changes involved reducing meat provided. During the sustainable food week only 1-3 meat and fish options were available at the salad bar as opposed to a regular 8-10 options.

Campaign: Every day different 'table talker' information were provided. The focus was on reducing meat and increasing vegetable consumption accompanied by information about resources needed to produce meat.

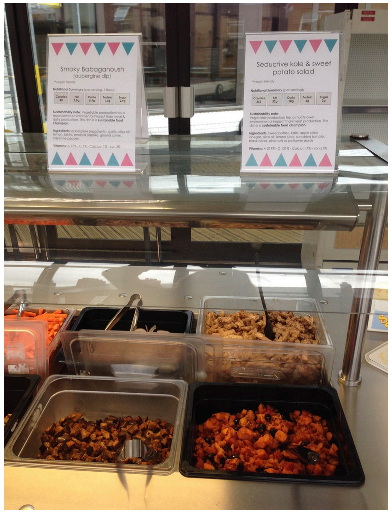

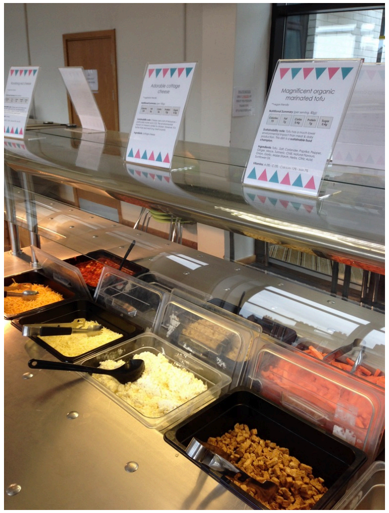

Workshop before the behaviour change intervention

We organised two workshops prior to the sustainable food week for the employees. During these workshops the employees could have a look at the proposed changes and comment on what they (dis)liked. The aim of the workshops was to increase acceptance of changes and include employees in the development.

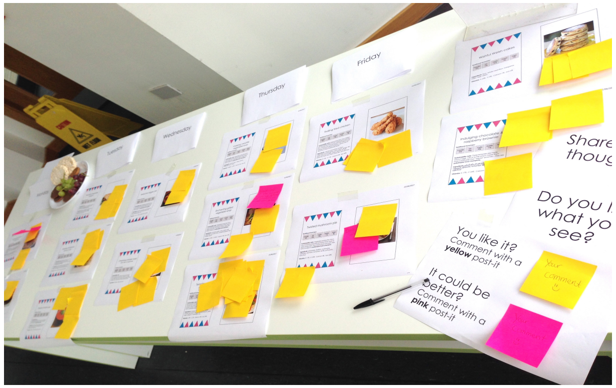

Picture of voting and feedback

Coloured post-its indicate employees (dis)likes for proposed menu.

Dish	likes (yellow post it's)	comments	dislikes (pink post-its)	comments
**salad bar**				
kale & sweet potato salad	8		0	
hummus	7	better than tescos!	0	
free range eggs	6		0	
sweetcorn	4		0	
tuna	4		0	
nutty rice pilaf	6		0	
tortilla chips	4		0	
salsa	5		0	
roasted vegetables	5		0	
lettuce	4		0	
rosemary butter beans	3		0	
beetroot	3		0	
mozzarella	4		0	
cucumber	3		0	
coronation chicken	4		1	no halal!
carrot fennel slaw	4	tasty | yes please!	0	
olives	2		0	
aubergine dip (smoky babaganoush)	3	I would definetly appreciate this	0	
onions	2	any chance of red	0	
red cheese	1		0	
butter benas	1	this would be a great alternative	0	
tomatoes	0		0	
other comments:	more variety, peppers,			

**premium meals**				
**Monday**				
big puff pie	6		0	
spaghetti bolognese (veg)	3		1	any other pasta but
**Tuesday**				
vegetable lasagne	6		0	
chickpea & quinoa curry	5		0	
tomato soup	5		0	
**Wednesday**				
chicken roast	4		0	
veg. chili	3		0	
**Thursday**				
versatile soup	2		0	
chicken parmigania	10	have a variation with Quorn	0	
wraps	4		1	Would love if the courgette & avocados were replaces
**Friday**				
Mushroom pie	2		1	Don't like
Fried chicken	4		0	
**Desserts**				
chocolate raspberry browni	5		0	
Welsh cakes	3		0	
